# Gut Microbiota Alteration and Its Modulation with Probiotics in Celiac Disease

**DOI:** 10.3390/biomedicines11102638

**Published:** 2023-09-26

**Authors:** Angela Saviano, Carmine Petruzziello, Mattia Brigida, Maria Rita Morabito Loprete, Gabriele Savioli, Alessio Migneco, Veronica Ojetti

**Affiliations:** 1 Department of Emergency Medicine, Fondazione Policlinico Universitario A. Gemelli, IRCCS, 00168 Rome, Italy; angela.saviano@policlinicogemelli.it (A.S.); alessio.migneco@policlinicogemelli.it (A.M.); 2 Department of Emergency Medicine, Ospedale San Carlo di Nancy, GVM Care and Research, 00165 Rome, Italy; 3 Gastroenterology Unit, Policlinico Universitario Tor Vergata, 00133 Rome, Italy; 4 Department of Emergency Medicine, Fondazione Policlinico San Matteo University Hospital, 27100 Pavia, Italy; gabrielesavioli@gmail.com; 5 Department of Emergency Medicine-Fondazione Policlinico Universitario A. Gemelli, Università Cattolica del Sacro Cuore, 00168 Rome, Italy

**Keywords:** celiac disease, probiotics, gut microbiota, immune system, gluten, diet

## Abstract

Celiac disease (CD) is a chronic inflammation of the small intestine triggered by gluten ingestion in genetically predisposed people. Recent literature studies highlight the possible role of the gut microbiota in the pathogenesis of this disease. The gut microbiota is a complex community of microorganisms that can interact with the innate and adaptative immune systems. A condition of dysbiosis, which refers to an alteration in the composition and function of the human gut microbiota, can lead to a dysregulated immune response. This condition may contribute to triggering gluten intolerance, favoring the development and/or progression of CD in genetically susceptible patients. Interestingly, studies on children and adults with CD showed a different microbiome profile in fecal samples, with a different degree of “activity” for the disease. From this point of view, our review aimed to collect and discuss modern evidence about the alteration of the gut microbiota and its modulation with probiotics, with possible future indications in the management of patients affected by CD.

## 1. Introduction

Celiac disease (CD) is a common autoimmune disorder of the gut, with an estimated prevalence of 0.5–1% in the general population [1]. It is characterized by a chronic inflammation of the small intestine, triggered by gluten ingestion in genetically predisposed people [2]. It involves not only genetic factors but also environmental ones, and it can be identified with a specific histological and serological profile. Gluten is a protein consumed in significant quantities by individuals. It is composed of gliadin (a protein rich in prolines and glutamines) and is not completely digested by gut enzymes. In predisposed people, because of a triggering event, such as an enteric infection, gliadin can activate the innate and adaptative immune systems with a break in the tolerance to this antigen. This, as reported by several literature studies, leads to an increase in gut permeability and a rise in exposure to gut pathogens and gut bacteria components (such as bacterial DNA, endotoxins, and metabolites) [3]. From this perspective, several studies have shown the role of the gut microbiota in the pathogenesis of CD with an association between CD and changes in microbiota composition. For example, HLA-DQ2+ infants (infants at high risk of CD) showed a higher abundance of *Firmicutes* and a decreased representation of *Bacteroidetes* in their fecal samples [4,5,6,7]. Further, they also had a decreased number of *Lactobacillus* species and an increased proportion of Gram-negative bacteria. Other studies confirmed that CD patients have a decreased number of *Lactobacillus*, *Bifidobacterium*, and *Bacteroides*. Interestingly, the higher incidence of Gram-negative bacteria was associated with the “symptomatic presentation” of the disease. Nowadays, an increased prevalence of symptomatic CD has been documented, and it can be associated with gut microbiota changes and environmental modification, too [8]. Research studies report that the interplay between host factors (such as gut microbiota composition, exposure to non-pathogenic microorganisms, etc.) and environmental factors can determine the onset of symptomatic CD. A gut microbiota composed of proinflammatory Gram-negative bacteria and a decreased number of Lactobacilli leads to the activation of the immune system, increased inflammation triggered by pathogen strains of bacteria, and the expression of epitopes that mimic gluten/gliadin by stimulating lymphocytes. Moreover, *Bacteroides* and *Escherichia coli* are associated with a rise in intestinal permeability, and the production of short-chain fatty acids (SCFAs) by some of these “gut bacteria” can also modify the regulation of intestinal barrier function together with the overall immune response to dietary antigens [3,9]. Different gut microbial communities between DQ2− and DQ2+ celiac patients have been found, with a prevalence of *Bacteroidetes* and *Enterococcus* and a decreased number of *Clostridium perfrigens*, *Parabacterioidetes*, and *Veillonella* in high-profile genetic-risk children. However, some studies have reported on the influence of the type of feeding and early antibiotic exposure on the modulation of the gut microbiota and the subsequent predisposition to develop CD [10,11,12]. Patients who underwent a gluten-free diet (which is associated with different microbiota in fecal samples) had a lower immune stimulatory effect on blood–mononuclear cells compared with patients on a regular gluten diet [1,5]. The gluten-free diet was proven to modify the composition and immune properties of the human gut microbiota with a decrease in the number of healthy gut bacteria and a concomitant increase in unhealthy bacteria because of a reduction in the intake of polysaccharides with the gluten-free diet. The gluten-free diet (GFD) is considered the mainstay treatment for patients with CD [13]. However, some gluten-free products have increased fat and sugar content and low levels of iron, magnesium, zinc, and vitamins; this can often result in a nutritional deficit. Thompson et al. [14] conducted a survey in which they showed that women on GFDs had a mean average intake of 46%, 44%, and 31% of their daily fiber, iron, and calcium intake requirements; in men, these values were doubled. In recent years, pseudocereals such as quinoa, amaranth, and other cereals have become popular, with high nutrient levels but higher costs than rice. In the United States, on patients with CD, recent studies have used a gluten-free cereal known as sorghum that contains flavones and tannins, with anti-inflammatory activity observed in preclinical studies [15].

Another point to emphasize is that different diets affect the health of gut flora. For example, polyphenols derived from whole grains play a beneficial role in the balance of gut microbiota, improving glucose homeostasis, lowering lipid peroxidation, and reducing oxidative stress [16]. Golfetto et al. [8] analyzed gut microflora in celiac patients on GFDs compared with healthy controls and found an imbalance in their intestinal gut microbiota with a decrease in *Lactobacilli* and *Bifidobacteri* and an increase in *Enterobacteriaceae*. This finding was mainly relatable to the reduced intake of polysaccharides. In this case, some bacteria use more dietary substrates compared with others in competition, but, on the other hand, those micronutrients modify the pH of the bile and may influence microbial growth [17]. Even so, we do not know if this imbalance was born before the GFD or whether it developed later. Since GFDs are very difficult to follow, alternatives are being sought to help patients comply. Biotechnological approaches (enzymatic therapy or modified grains) are currently being explored to obtain safer and healthier foods for celiacs and produce “detoxified” gluten with nutritional and organoleptic properties superior to those of gluten-free products currently on the market [15,17], but further research is needed to explore this field. Meanwhile, substances such as probiotics, which have proven beneficial for other gastrointestinal diseases, are under investigation in patients with CD [18].

## 2. The Role of Gut Microbiota and Its Modulation with Probiotics in Patients with Celiac Disease

The human gut microbiota regulates many functions of the organism, such as nutrition, digestion, and immune function. As described above, the HLA-DQ genotype may influence the gut microbiota composition, and patients with CD may present with alterations of it, thus influencing immune function, the maintenance of gut barrier integrity, the absorption of nutrients and metabolites, the homeostasis between innate and adaptative immune responses, and the inhibition of the growth of pathogens. Evidence underlines that, in patients with CD, soluble CD14, an indicator of the activation of innate immune cells in response to the mucosal translocation of Gram-negative bacteria, is increased [19]. Furthermore, *Lactobacilli* and *Bifidobacterium* may play a role in the breakdown of gluten and its peptides, modifying their immunogenicity and promoting the development of CD [19]. Several studies have described how *Bifidobacteria* strains are also able to reduce the epithelial permeability triggered by gluten, while other species, such as *Escherichia coli*, may have a protective effect on gut barrier integrity. *Lactobacilli*, too, have shown immunomodulatory properties with an influence on immune responses to gluten [19]. Studies on infants [20] have shown that infants with a high genetic risk of CD have gut microbiota comprising higher proportions of *Proteobacteria* and *Firmicutes* and lower proportions of *Bifidobacteria* and *Actinobacteria*. Other studies [21,22] have reported that milk feeding plus the HLA-DQ genotype has an impact on infant gut microbiota compositions. Gut bacteria such as the Lactobacilli and Bifidobacterium strains may have some advantages in patients with a high risk of CD. They can break down the immune response to gluten and its peptides. Caminero et al. [1] demonstrated that Lactobacilli can detoxify gliadin after digestion by human proteases and that immunogenic peptides produced by *Pseudomonas aeruginosa* proteases were degraded and were less immunogenic in the presence of Lactobacillus. Literature studies have shown that patients with CD and gastrointestinal symptoms have lower levels of *Faecalibacterium prausnitzii* than healthy controls [19]; while it is a good representation of *Escherichia coli*, *Streptococcus thermophilus* may have a protective effect on gut barrier function with anti-inflammatory effects [23]. These conclusions suggest that specific microbial strains could influence gluten-processing activities and be used as adjunctive therapy for CD [16], and alterations in the gut microbiota composition could be responsible for the manifestation of CD and a more pronounced active phase of the disease [23], with a possible evolution. In fact, the literature [24] has shown that a prevalence of *Bacteroides* species in patients with CD may contribute to the degradation of mucins and increased intestinal permeability and, similarly, an abundance of some *Staphylococcus* and *Enterococcus* strains [24]. *Pseudomonas aeruginosa*, too, can cause increased gut inflammation during gluten exposition. Several mechanisms have been hypothesized to explain this relationship between alterations in gut microbiota and the manifestation of CD. Infections, feeding types, antibiotic use, environmental factors, and genetic factors in combination with the microbiota itself (for the production of immunogenic gluten peptides; epitopes that mimic gliadin; the stimulation of proinflammatory immune systems that release damaging cytokines for gut barrier integrity) may contribute to pathogenesis and the active phase of CD [22,23,24,25].

**Figure 1:**
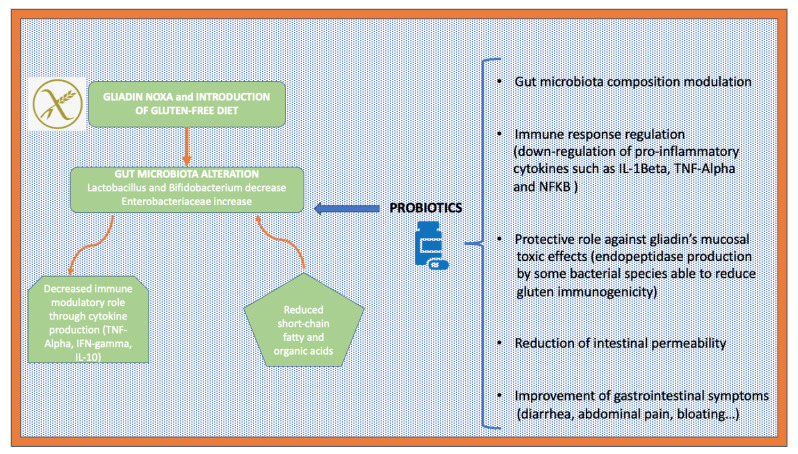
Schematic representation of the potential benefits of probiotic use in patients affected by celiac disease.

From this point of view, strategies meant to rebalance the intestinal microbiota can play a key role. In fact, the use of probiotics could have some advantageous effects on celiac patients (Figure 1). Literature studies have reported that probiotics could regulate the immune response, reduce inflammatory reactions, restore beneficial bacteria in the gut, produce bacteriocins against pathogens, and reduce diarrhea symptoms in celiac patients. The effects of probiotics have been mainly studied regarding “typical” celiac symptoms (the effects on “atypical” ones in CD patients are not understood yet). Studies on HLA DQ8+ mice (immunized with gliadin) underline that some strains, such as *Lactobacillus paracasei*, *Lactobacillus fermentum*, and *Bifidobacterium lactis*, have the ability to induce the production of TNF-alpha with an “inductive” effect on innate and adaptive immunity [24,25]. Others have reported that *Bifidobacterium longum CECT 7347* can decrease the production of inflammatory cytokines and the production of CD 4+ T cells in mouse models; furthermore, it ameliorates gliadin-induced enteropathy. *Bifidobacterium breve* prevents intestinal inflammation through the modulation of T cells and Tregs and the induction of IL-10. *Saccharomyces boulardii KK1* improves enteropathy in animal models of CD and decreases the production of inflammatory cytokines. *Lactobacillus casei* was found to be effective in restoring the normal gut mucosa architecture; finally, studies on *Lactobacillus rhamnosus GG* have reported that this probiotic could protect the intestinal mucosa from damage induced by the immune response to gliadin. Many other studies are needed to better understand the role of gut microbiota in CD pathogenesis and the therapeutic approach and target needed to prevent autoimmunity phenomena and reestablish tolerance.

## 3. Potential Benefits of Probiotic Use in Children with Celiac Disease

In recent years, several randomized controlled trials (RCTs), open-label trials, clinical studies, and pilot studies have been conducted to explore the effects of probiotics on various outcomes in children with CD. The diagnosis of CD is based on clinical, serological, and histological criteria. Research studies often include children in different stages of the disease, such as newly diagnosed patients, those on a gluten-free diet, and those with persistent symptoms despite adherence to a gluten-free diet. Clinical trials assessing the effects of probiotics on gastrointestinal symptoms in children with CD have provided valuable insights into the potential benefits of probiotic supplementation.

In a review by Tremblay et al. in 2021 [26], the researchers aimed to evaluate the efficacy of a multi-strain probiotic in children with CD who experienced persistent symptoms despite adhering to a gluten-free diet. The study enrolled a group of children and randomly assigned them to receive either a probiotic or a placebo for a specified duration. The results of the trial revealed a significant improvement in gastrointestinal symptoms (reductions in abdominal pain and bloating and improvements in stool consistency) in the probiotic group compared with the placebo group. These findings suggest that the multi-strain probiotic may help alleviate the discomfort associated with gastrointestinal symptoms in children with celiac disease, even when they are adhering to a gluten-free diet.

Similarly, a trial conducted by Ali et al. in 2022 [18] demonstrated that probiotic intervention led to a significant reduction in abdominal pain and bloating compared with baseline measurements. This finding suggests that the specific probiotic strain used in the study may have potential benefits in alleviating gastrointestinal symptoms in children with newly diagnosed CD. Moreover, a significant reduction in intestinal permeability in patients with CD treated with a probiotic compared with a placebo has been shown by other research studies [18,27]. This finding suggests that the probiotic intervention may help restore intestinal barrier function, leading to decreased permeability and the reduced passage of harmful substances across the intestinal lining.

Another pilot study by Demiroren et al. in 2020 [28] explored the effects of a synbiotic, a combination of probiotics and prebiotics, on gut barrier function in children with celiac disease. The study aimed to determine whether the synbiotic intervention could improve gut barrier integrity by influencing the expression of tight junction proteins, which play a crucial role in maintaining the integrity of the intestinal barrier. The results of the pilot study showed promising outcomes, with an improvement in gut barrier integrity demonstrated by the increased expression of tight junction proteins in the group receiving the synbiotic intervention. In addition to their effects on gut barrier function and intestinal permeability, probiotics have also been investigated for their impact on immunological markers in children with celiac disease. The immune response to gluten plays a central role in the pathogenesis of celiac disease, and modulating this response is of significant interest in therapeutic interventions.

A randomized controlled trial conducted by Klemenak et al. in 2015 [29] focused on evaluating the impact of a specific probiotic strain on proinflammatory cytokine levels in children with celiac disease who were following a gluten-free diet. The study aimed to determine whether probiotics could influence the immune response to gluten and potentially reduce inflammation in this population. The participants were randomly assigned to either a probiotic group or a placebo group, and their proinflammatory cytokine levels were measured before and after the intervention period. The results of the trial revealed that the probiotic group exhibited a significant reduction in proinflammatory cytokines compared with the placebo group. These findings suggest that probiotics may have an immunomodulatory effect, potentially mitigating the immune response to gluten and alleviating inflammation in children with celiac disease. The precise mechanisms by which probiotics exert their effects on the immune response in celiac disease are still not fully understood and require further investigation. It is likely that probiotics modulate the immune system through interactions with the gut microbiota, as well as by directly influencing immune cells and cytokine production. Understanding these mechanisms will provide valuable insights into the potential therapeutic benefits of probiotics in managing immune responses in children with celiac disease. The results suggest that probiotic interventions can lead to a significant reduction in proinflammatory cytokines, highlighting their potential immunomodulatory effects. Additionally, the long-term implications of probiotic interventions on the immune responses and clinical outcomes of children with celiac disease need to be further investigated. However, more research is necessary to elucidate the underlying mechanisms and establish standardized protocols for probiotic use in managing the immune responses of children with celiac disease. Long-term studies are also needed to determine the sustained effects and clinical implications of probiotic interventions in this population. Adherence to a strict gluten-free diet is the cornerstone of managing celiac disease and preventing gluten-induced damage to the intestinal lining. However, maintaining long-term dietary adherence can be challenging, particularly for children, who may face social and practical obstacles. As a result, researchers have conducted clinical trials to explore whether probiotics can improve adherence to a gluten-free diet in children with celiac disease.

## 4. Potential Benefits from Probiotic Use in Adult Patients with Celiac Disease

Probiotics can have a protective role against the toxic effects of gliadin, and they can decrease intestinal permeability and lead to the improvement of gastrointestinal symptoms [5,30] because of their ability to modulate both gut microbiota composition and immune responses. Regarding the modulation of gut microbiota composition in patients with CD, it is necessary to highlight some elements regarding the intestinal dysbiosis that develops in these subjects before and after diagnosis [31,32,33]. Evidence shows that *Lactobacilli*, *Bifidobacteria,* some species of *Clostridium* (such as *Clostridium histolyticum* and *Clostridium lituseburense*), and *Fecalibacterium prausnitzi* are substantially reduced in patients with CD. On the other hand, a greater concentration of *Bacteroides* and *Escherichia coli* (*E. coli*) and an increase in the Gram(−)/Gram(+) ratio have been detected in the fecal microbiota of patients with CD [31,32,34]. Sanz and colleagues [34] documented how, in celiac patients, the proportion of *Bifidobacteria* and Lactobacilli was reduced, while the proportion of *Enterobacteriaceae* increased. A possible explanation of the dysbiosis that develops in these patients could stem from the fact that gluten exerts a prebiotic action, and its absence during a gluten-free diet induces variations in the intestinal microbiota even in healthy subjects [35]. Indeed, foods such as chicory roots, raw garlic, and wheat are rich in prebiotics, i.e., substances that induce the growth or activity of microorganisms (especially *Bifidobacteria* and *Lactobacilli*) and, therefore, contribute to the well-being of the host. Moreover, one of the current research lines is focused on the possibility of enriching gluten-free foods with prebiotics. The addition of inulin to gluten-free bread has been proven to indirectly increase the absorption of calcium and other nutrients by modifying the altered gut microbiota [5,36]. For example, Wacklin et al. [37] showed that intestinal dysbiosis is linked to iron deficiency, reduced bone density, anemia, and refractory gastrointestinal symptoms in patients on a gluten-free diet. As a matter of fact, there are many theories on how this dysbiosis could facilitate a loss of tolerance to gluten in genetically predisposed subjects, thus increasing the permeability of the intestinal mucosa with leakage from the tight junctions during inflammation and greater recruitment of T cells [30,38,39]. An interesting study by Joelson et al. [40], recently published, starts with the assumption that dysbiosis in celiac patients leads to the greater use of probiotics, and, in this prospective study, the authors evaluated patients with celiac disease who answered a questionnaire regarding demographics, diagnosis, symptoms, and treatment. In this protocol, probiotic users were compared with non-probiotic users, and, through multivariate analysis, it was noted that about one-third of celiac patients reported the use of probiotics. The predictive factors of this increased use of probiotics were found to be the age at diagnosis, the persistence of symptoms despite a gluten-free diet, and not having received sufficient information at the time of diagnosis about nutrition and the disease. The authors also showed that patients over the age of fifty used probiotics up to twice as much as younger patients.

### 4.1. Role of Probiotics and Regulation of the Immune Response

Another aspect to analyze regarding the role of probiotics in adult patients with celiac disease is the regulation of the immune response and its protective role against the toxic effects of gliadin [41,42]. In fact, some bacterial species from the intestinal microbiota, such as *Bifidobacterium* and *Lactobacillus*, produce endopeptidases that are able to digest the epitopes of gluten and, therefore, reduce its immunogenicity [43]. To understand the role of some bacterial clusters in the modulation of the immune response, De Palma et al. [22] evaluated the effects of *Bifidobacterium bifidum* and *Bifidobacterium longum* alone or with celiac disease triggers on peripheral-blood mononuclear cells, comparing these effects with those of Gram-negative bacteria such as *E. coli* and *Bacteroides fragilis*. In this study, it was noted that Gram-negative bacteria induced a greater Th-1 secretion response to proinflammatory cytokines and the activation of other mechanisms such as IFN-c, IL-12, HLA-DR, and CD40 compared with *Bifidobacteria* species [21]. On the other hand, the latter increased the expression of CD83, which is a marker of mature dendritic cells. The authors, therefore, concluded that the microbiota could regulate monocytes and the IFN reaction to gliadin locally [21]. Another study by Laparra and colleagues [44] evaluated other strains of *Bifidobacteria*, which also are able to improve the altered bacterial composition of patients with celiac disease by reducing inflammation. These authors noted how digested gliadin fragments and *Bifidobacteria,* in particular, *Bifidobacterium longum*, induced the down-regulation of proinflammatory cytokines such as IL-1Beta, TNF-Alpha, and NFKB. Furthermore, Fallani et al. [45] analyzed the potential role of a probiotic composition, VSL#3 (*Bifidobacterium breve, B longum, B infantis, Lactobacillus plantarum*, *Lactobacillus acidophilus, Lactobacillus casei, Lactobacillus delbrueckii bulgaricus,* and *Streptococcus thermophiles*) in reducing the toxic properties of wheat and found that this composition was highly effective in hydrolyzing gliadin peptides, reducing immune responses.

### 4.2. Probiotics, Gut Permeability, and Gastrointestinal Symptoms

A randomized, double-blind, placebo-controlled study [46] evaluated *B. infantis* and its effects on gut permeability, symptoms, and the presence of inflammatory cytokines in patients with untreated celiac disease. The authors demonstrated that the administration of this probiotic was unable to modify the gut barrier function. In any case, an important improvement in dyspeptic symptoms and a reduction in constipation were registered; it was also found that the scores concerning abdominal pain and diarrheal symptoms were reduced, although not significantly, and there was no difference in terms of inflammatory markers between the probiotic group and the placebo group. Once again, intestinal dysbiosis may be responsible for the alteration of intestinal permeability in celiac patients through the destruction of tight junctions and an alteration in the balance between proinflammatory and anti-inflammatory responses in favor of the up-regulation of T-helper cells, type 127 [45]. In fact, a study conducted on a mouse model [25] demonstrated how the administration of *Lactobacillus casei* can lead to the recovery of gut homeostasis and the restoration of the mucosal structure. However, there are not enough in vivo studies in the literature to demonstrate this restoration of mucosal architecture, and indeed, there is different evidence that underlines how supplementation with probiotics cannot replace a gluten-free diet [22,47]. Finally, most of the studies in the scientific literature have evaluated what the benefits could be for the symptoms of celiac patients after the administration of probiotics. A recently published study by Francavilla et al. [38] prospectively and randomly evaluated a mix of probiotics composed of *Lactobacillus casei*, *Lactobacillus plantarum*, *Bifidobacterium animalis*, and two strains of *Bifidobacterium breve*. This mixture of probiotics, administered daily for 6 weeks, proved effective in improving the severity of gastrointestinal symptoms; moreover, these authors demonstrated modifications to the composition of the microbiota up to 6 weeks after the interruption of the supplementation. In a systematic review and meta-analysis conducted by Seiler and colleagues [48] a few years ago, the authors identified six randomized clinical trials (four on celiac adults and two on celiac children) and found that probiotic supplementation ameliorated GI symptoms when these were assessed using the GI symptom rating scale questionnaire, while no difference was noted when other questionnaires were used. The authors also concluded that the overall certainty of evidence ranged from very low to low, that there was no difference in adverse events registered in the placebo vs. probiotic groups, and that there were insufficient data on an overall improvement in quality of life. One more recent systematic review and meta-analysis by Mozafarybazargany et al. [43] on clinical trials gathered data on probiotic use in celiac disease from more than 800 patients, about 200 of whom were adults. The authors confirmed no difference in adverse events compared with the placebo and provided further support to the idea that probiotics might alleviate gastrointestinal symptoms in this category of patients. However, these authors stressed the importance of increasing the level of evidence using new clinical trials. On the other hand, there is no evidence to date that probiotic use can prevent the onset of celiac disease or that it can replace a gluten-free diet [22,43,47]. Furthermore, microbiota composition is known to vary geographically because of genetic variability between different populations [49]. Therefore, not only the number and quality of clinical trials need to be strengthened but the best combination of probiotics might also have to be population-personalized and, possibly, individually personalized for adults and children with celiac disease in the future.

## 5. Postbiotics and Celiac Disease

Postbiotics have recently emerged as another group of beneficial compounds that can help improve health. Postbiotics are also called “ghost probiotics” and are defined as a “preparation of inanimate microorganisms and/or their components that confer a health benefit on the host”. They are bioactive compounds that probiotic gut bacteria produce when they consume prebiotics or products secreted by living bacteria or that are released after their lysis [50]. Examples of postbiotics are molecules such as SCFAs, which help healthy bacteria flourish; lactic acid; and bioactive peptides. This definition can also be extended to protein compounds, hydrogen peroxide (H_2_O_2_), bacteriocins, organic acids, exopolysaccharides, and enzymes [51]. Even though a postbiotic can be considered a waste product of probiotic bacteria, it shows some health benefits. Its beneficial effect on microbiota is carried out through the improvement of epithelial barrier function, the modulation of immune responses, and systemic metabolism or signaling via the nervous system. The two major mechanisms are the modulation of the immune system by stimulating various cytokines and immune modulators’ expression, enhancing the function of the gut barrier through the stimulation of tight junctions, and mucus production. Postbiotics, compared with probiotics, have some advantages: first, higher stability and safety; second, they are not living bacteria, so there is no risk of microbial infection or translocation. Nevertheless, the exact mechanisms of action and, thus, the relative molecular pathways leading to gut improvement are still unknown. Regarding the possible positive effect of postbiotics on CD, there have been some experiments in vitro on Caco-2 cells. A study by Conte and coworkers [52] showed the beneficial postbiotic effect of *Lactobacillus paracasei CBA L74* in preventing the gliadin-induced activation of inflammatory responses. In another in vitro model of organoids from celiac and non-celiac patients, Freire et al. [53] used postbiotic butyrate, lactate, and polysaccharide A from *B. fragilis* and demonstrated that they can reduce leaky gut through the increased expression of the tight junction sealing the molecule claudin-18. Moreover, butyrate and lactate can reduce the induced immune activation of celiac disease organoids pre-treated with gliadin. The gastrointestinal proteases are able, both in vitro and/or in vivo, to degrade and inactivate gluten, thus reducing the number of gluten peptides reaching the small intestine. Peptidases can be obtained from probiotic preparations involving lactobacilli or other microorganisms, such as the *B. tequilensis* strain. Recently, preprocessed foods that reduce the gluten load and its toxicity have been marketed. In particular, in this process, prolyl endopeptidase obtained using *Aspergillus niger* or proteolytic enzyme obtained from the papaya plant and papain are used to reduce gluten concentration. However, none of these peptidases break down gluten completely, so they are not yet used as treatments for celiac disease. Other prolyl endopeptidases from *Flavobacterium meningosepticum*, *Sphingomonas capsulate*, and *Myxococcus xanthus* degrade immunogenic gluten amino acid sequences, reducing its toxicity in celiac patients. In one clinical study [50], a bacterial endopeptidase called TAK-062 showed a high capacity for gluten hydrolysis, and after its ingestion, around 97–99% of the gluten was degraded. Postbiotics represent a promising new therapeutic approach to treating celiac patients, even if they are not yet available.

## 6. Fecal Transplant in Patients with Celiac Disease

Recent studies have started to investigate the use of fecal transplants in the management of CD [54,55]. Studies led by Collado and colleagues [56,57] demonstrated differences between fecal microbiota in children and adults with CD and healthy controls (a higher abundance of *Bacteroides, Clostridium*, and *Staphylococcus* and higher counts of *Clostridium histolyticum* and *Eubacterium rectale*). The selection of donors and the procedure itself are very complex, and there are not enough studies to demonstrate the safety and efficiency of fecal transplants in patients affected by CD. To date, few case reports have been published. In a case report on a patient with type II refractory celiac disease and recurrent *Clostridioides difficile* infection, the FMT was successful. The procedure cured the *Clostridioides difficile* infection and mitigated CD symptoms. At a follow-up after six months, no villous atrophy in duodenal biopsies was present [55]. As reported by other literature studies, FMTs have some risks; in fact, an inappropriate donor selection could expose a recipient to the dysbiosis status of the gut microbiota, worsening abdominal symptoms [58,59,60,61], and this might apply even in the case of CD. Therefore, much more research and studies are needed to explore this field.

## 7. Conclusions

In conclusion, the gut microbiota plays a key role in the pathogenesis of CD. Several factors can influence the health of the gut microbiota, such as diet. The mechanisms through which the gut microbiota modulates the immune system’s response to gluten ingestion are not fully known, and neither is the characterization of the gut microbiota in celiac patients. Despite this, it is known that a balanced gut microbiota composition can play a key role in the prevention, onset, and treatment of CD. Therefore, these observations may lead to new therapies and nutritional strategies for the management of these patients, ranging from biotechnological approaches involving gluten protein detoxification with enzymatic therapy and/or modified grains to supplementation with probiotics. More studies are, however, required to deepen this topic.

## Data Availability

Not applicable.

## References

[1] Caminero A., Verdu E.F. (2019). Celiac disease: Should we care about microbes?. Am. J. Physiol. Gastrointest. Liver Physiol..

[2] Tarar Z.I., Zafar M.U., Farooq U., Basar O., Tahan V., Daglilar E. (2021). The Progression of Celiac Disease, Diagnostic Modalities, and Treatment Options. J. Investig. Med. High. Impact Case Rep..

[3] Verdu E.F., Galipeau H.J., Jabri B. (2015). Novel players in coeliac disease pathogenesis: Role of the gut microbiota. Nat. Rev. Gastroenterol. Hepatol..

[4] Cenit M.C., Codoñer-Franch P., Sanz Y. (2016). Gut Microbiota and Risk of Developing Celiac Disease. J. Clin. Gastroenterol..

[5] Krishnareddy S. (2019). The Microbiome in Celiac Disease. Gastroenterol. Clin. N. Am..

[6] Chibbar R., Dieleman L.A. (2019). The Gut Microbiota in Celiac Disease and probiotics. Nutrients.

[7] Wu X., Qian L., Liu K., Wu J., Shan Z. (2021). Gastrointestinal microbiome and gluten in celiac disease. Ann. Med..

[8] Golfetto L., de Senna F.D., Hermes J., Beserra B.T., França Fda S., Martinello F. (2014). Lower bifidobacteria counts in adult patients with celiac disease on a gluten-free diet. Arq. Gastroenterol..

[9] Cenit M.C., Olivares M., Codoñer-Franch P., Sanz Y. (2015). Intestinal Microbiota and Celiac Disease: Cause, Consequence or Co-Evolution?. Nutrients.

[10] Cristofori F., Indrio F., Miniello V.L., De Angelis M., Francavilla R. (2018). Probiotics in Celiac Disease. Nutrients.

[11] Dargenio V.N., Castellaneta S., Panico S., Papagni M.E., Dargenio C., Schettini F., Francavilla R., Cristofori F. (2022). Probiotics and gastrointestinal diseases. Minerva Pediatr..

[12] Valitutti F., Leonard M.M., Kenyon V., Montuori M., Piemontese P., Francavilla R., Malamisura B., Norsa L., Calvi A., Lionetti M. (2023). Early Antibody Dynamics in a Prospective Cohort of Children At Risk of Celiac Disease. Am. J. Gastroenterol..

[13] Aljada B., Zohni A., El-Matary W. (2021). The Gluten-Free Diet for Celiac Disease and Beyond. Nutrients.

[14] Thompson T., Dennis M., Higgins L.A., Lee A.R., Sharrett M.K. (2005). Gluten-free diet survey: Are Americans with coeliac disease consuming recommended amounts of fibre, iron, calcium and grain foods?. J. Hum. Nutr. Diet..

[15] Pontieri P., Mamone G., De Caro S., Tuinstra M.R., Roemer E., Okot J., De Vita P., Ficco D.B., Alifano P., Pignone D. (2013). Sorghum, a healthy and gluten-free food for celiac patients as demonstrated by genome, biochemical, and immunochemical analyses. J. Agric. Food Chem..

[16] Dong L., Qin C., Li Y., Wu Z., Liu L. (2022). Oat phenolic compounds regulate metabolic syndrome in high fat diet-fed mice via gut microbiota. Food Biosci..

[17] Kaliciak I., Drogowski K., Garczyk A., Kopeć S., Horwat P., Bogdański P., Stelmach-Mardas M., Mardas M. (2022). Influence of Gluten-Free Diet on Gut Microbiota Composition in Patients with Coeliac Disease: A Systematic Review. Nutrients.

[18] Ali B., Khan A.R. (2022). Efficacy of Probiotics in Management of Celiac Disease. Cureus.

[19] Pecora F., Persico F., Gismondi P., Fornaroli F., Iuliano S., De’Angelis G.L., Esposito S. (2020). Gut Microbiota in Celiac Disease: Is There Any Role for Probiotics?. Front. Immunol..

[20] Olivares M., Benítez-Páez A., de Palma G., Capilla A., Nova E., Castillejo G., Varea V., Marcos A., Garrote J.A., Polanco I. (2018). Increased prevalence of pathogenic bacteria in the gut microbiota of infants at risk of developing celiac disease: The PROFICEL study. Gut Microbes.

[21] Olivares M., Flor-Duro A., Sanz Y. (2021). Manipulation of the gut microbiome in gluten-intolerance. Curr. Opin. Clin. Nutr. Metab. Care.

[22] De Palma G., Cinova J., Stepankova R., Tuckova L., Sanz Y. (2010). Pivotal Advance: Bifidobacteria and Gram-negative bacteria differentially influence immune responses in the proinflammatory milieu of celiac disease. J. Leukoc. Biol..

[23] Leonard M.M., Valitutti F., Karathia H., Pujolassos M., Kenyon V., Fanelli B., Troisi J., Subramanian P., Camhi S., Colucci A. (2021). Microbiome signatures of progression toward celiac disease onset in at-risk children in a longitudinal prospective cohort study. Proc. Natl. Acad. Sci. USA.

[24] Rossi R.E., Dispinzieri G., Elvevi A., Massironi S. (2023). Interaction between Gut Microbiota and Celiac Disease: From Pathogenesis to Treatment. Cells.

[25] D’Arienzo R., Stefanile R., Maurano F., Mazzarella G., Ricca E., Troncone R., Auricchio S., Rossi M. (2011). Immunomodulatory effects of Lactobacillus casei administration in a mouse model of gliadin-sensitive enteropathy. Scand. J. Immunol..

[26] Tremblay A., Xu X., Colee J., Tompkins T.A. (2021). Efficacy of a Multi-Strain Probiotic Formulation in Pediatric Populations: A Comprehensive Review of Clinical Studies. Nutrients.

[27] Drabińska N., Krupa-Kozak U., Jarocka-Cyrta E. (2020). Intestinal Permeability in Children with Celiac Disease after the Administration of Oligofructose-Enriched Inulin into a Gluten-Free Diet-Results of a Randomized, Placebo-Controlled, Pilot Trial. Nutrients.

[28] Demiroren K. (2020). Can a Synbiotic Supplementation Contribute to Decreasing Anti-Tissue Transglutaminase Levels in Children with Potential Celiac Disease?. Pediatr. Gastroenterol. Hepatol. Nutr..

[29] Klemenak M., Dolinšek J., Langerholc T., Di Gioia D., Mičetić-Turk D. (2015). Administration of Bifidobacterium breve Decreases the Production of TNF-α in Children with Celiac Disease. Dig. Dis. Sci..

[30] Marasco G., Cirota G.G., Rossini B., Lungaro L., Di Biase A.R., Colecchia A., Volta U., De Giorgio R., Festi D., Caio G. (2020). Probiotics, Prebiotics and Other Dietary Supplements for Gut Microbiota Modulation in Celiac Disease Patients. Nutrients.

[31] Volta U., Caio G., Giancola F., Rhoden K.J., Ruggeri E., Boschetti E., Stanghellini V., De Giorgio R. (2016). Features and Progression of Potential Celiac Disease in Adults. Clin. Gastroenterol. Hepatol..

[32] Harnett J., Myers S.P., Rolfe M. (2016). Probiotics and the Microbiome in Celiac Disease: A Randomised Controlled Trial. Evid. Based Complement. Alternat Med..

[33] Ciacci C., Ciclitira P., Hadjivassiliou M., Kaukinen K., Ludvigsson J.F., McGough N., Sanders D.S., Woodward J., Leonard J.N., Swift G.L. (2015). The gluten-free diet and its current application in coeliac disease and dermatitis herpetiformis. United Eur. Gastroenterol. J..

[34] Sanz Y. (2010). Effects of a gluten-free diet on gut microbiota and immune function in healthy adult humans. Gut Microbes.

[35] Di Cagno R., Rizzello C.G., Gagliardi F., Ricciuti P., Ndagijimana M., Francavilla R., Guerzoni M.E., Crecchio C., Gobbetti M., De Angelis M. (2009). Different fecal microbiotas and volatile organic compounds in treated and untreated children with celiac disease. Appl. Environ. Microbiol..

[36] Capriles V.D., Arêas J.A. (2013). Effects of prebiotic inulin-type fructans on structure, quality, sensory acceptance and glycemic response of gluten-free breads. Food Funct..

[37] Wacklin P., Kaukinen K., Tuovinen E., Collin P., Lindfors K., Partanen J., Mäki M., Mättö J. (2013). The duodenal microbiota composition of adult celiac disease patients is associated with the clinical manifestation of the disease. Inflamm. Bowel Dis..

[38] Francavilla R., Cristofori F., Vacca M., Barone M., De Angelis M. (2020). Advances in understanding the potential therapeutic applications of gut microbiota and probiotic mediated therapies in celiac disease. Expert. Rev. Gastroenterol. Hepatol..

[39] Francavilla A., Ferrero G., Pardini B., Tarallo S., Zanatto L., Caviglia G.P., Sieri S., Grioni S., Francescato G., Stalla F. (2023). Gluten-free diet affects fecal small non-coding RNA profiles and microbiome composition in celiac disease supporting a host-gut microbiota crosstalk. Gut Microbes.

[40] Joelson A.M., Choy A.M., Blackett J.W., Molinsky R., Geller M.G., Green P.H., Lebwohl B. (2021). Probiotic Use in Celiac Disease: Results from a National Survey. J. Gastrointestin Liver Dis..

[41] Giorgi A., Cerrone R., Capobianco D., Filardo S., Mancini P., Zanni F., Fanelli S., Mastromarino P., Mosca L. (2020). A Probiotic Preparation Hydrolyzes Gliadin and Protects Intestinal Cells from the Toxicity of Pro-Inflammatory Peptides. Nutrients.

[42] Orlando A., Linsalata M., Notarnicola M., Tutino V., Russo F. (2014). Lactobacillus GG restoration of the gliadin induced epithelial barrier disruption: The role of cellular polyamines. BMC Microbiol..

[43] Mozafarybazargany M., Khonsari M., Sokoty L., Ejtahed H.S., Qorbani M. (2023). The effects of probiotics on gastrointestinal symptoms and microbiota in patients with celiac disease: A systematic review and meta-analysis on clinical trials. Clin. Exp. Med..

[44] Laparra J.M., Olivares M., Gallina O., Sanz Y. (2012). Bifidobacterium longum CECT 7347 modulates immune responses in a gliadin-induced enteropathy animal model. PLoS ONE.

[45] Fallani M., Young D., Scott J., Norin E., Amarri S., Adam R., Aguilera M., Khanna S., Gil A., Edwards C.A.A. (2010). Intestinal microbiota of 6-week-old infants across Europe: Geographic influence beyond delivery mode, breast-feeding, and antibiotics. J. Pediatr. Gastroenterol. Nutr..

[46] Smecuol E., Hwang H.J., Sugai E., Corso L., Cherñavsky A.C., Bellavite F.P., González A., Vodánovich F., Moreno M.L., Vázquez H. (2013). Exploratory, randomized, double-blind, placebo-controlled study on the effects of Bifidobacterium infantis natren life start strain super strain in active celiac disease. J. Clin. Gastroenterol..

[47] Al-Toma A., Volta U., Auricchio R., Castillejo G., Sanders D.S., Cellier C., Mulder C.J., Lundin K.E.A. (2019). European Society for the Study of Coeliac Disease (ESsCD) guideline for coeliac disease and other gluten-related disorders. United Eur. Gastroenterol. J..

[48] Seiler C.L., Kiflen M., Stefanolo J.P., Bai J.C., Bercik P., Kelly C.P., Verdu E.F., Moayyedi P., Pinto-Sanchez M.I. (2020). Probiotics for Celiac Disease: A Systematic Review and Meta-Analysis of Randomized Controlled Trials. Am. J. Gastroenterol..

[49] Johnson T.C., Diamond B., Memeo L., Negulescu H., Hovhanissyan Z., Verkarre V., Rotterdam H., Fasano A., Caillatzucman S., Grosdidier E. (2004). Relationship of HLA-DQ8 and severity of celiac disease: Comparison of New York and Parisian cohorts. Clin. Gastroenterol. Hepatol..

[50] Pultz I.S., Hill M., Vitanza J.M., Wolf C., Saaby L., Liu T., Winkle P., Leffler D.A. (2021). Gluten Degradation, Pharmacokinetics, Safety, and Tolerability of TAK-062, an Engineered Enzyme to Treat Celiac Disease. Gastroenterology.

[51] Raju S.A., Sanders D.S., Penny H.A. (2021). Coeliac Disease and Probiotics: Clinicians Need to Provide the Evidence Base for this Unmet Need. J. Gastrointestin Liver Dis..

[52] Conte M., Nigro F., Porpora M., Bellomo C., Furone F., Budelli A.L., Nigro R., Barone M.V., Nanayakkara M. (2022). Gliadin Peptide P31-43 Induces mTOR/NFkβ Activation and Reduces Autophagy: The Role of *Lactobacillus paracasei* CBA L74 Postbiotc. Int. J. Mol. Sci..

[53] Freire R., Ingano L., Serena G., Cetinbas M., Anselmo A., Sapone A., Sadreyev R.I., Fasano A., Senger S. (2019). Human gut derived-organoids provide model to study gluten response and effects of microbiota-derived molecules in celiac disease. Sci. Rep..

[54] Bibbò S., Abbondio M., Sau R., Tanca A., Pira G., Errigo A., Manetti R., Pes G.M., Dore M.P., Uzzau S. (2020). Fecal Microbiota Signatures in Celiac Disease Patients With Poly-Autoimmunity. Front. Cell Infect. Microbiol..

[55] van Beurden Y.H., van Gils T., van Gils N.A., Kassam Z., Mulder C.J., Aparicio-Pagés N. (2016). Serendipity in Refractory Celiac Disease: Full Recovery of Duodenal Villi and Clinical Symptoms after Fecal Microbiota Transfer. J. Gastrointestin Liver Dis..

[56] Collado M.C., Donat E., Ribes-Koninckx C., Calabuig M., Sanz Y. (2009). Specific duodenal and faecal bacterial groups associated with paediatric coeliac disease. J. Clin. Pathol..

[57] Collado M.C., Donat E., Ribes-Koninckx C., Calabuig M., Sanz Y. (2008). Imbalances in faecal and duodenal Bifidobacterium species composition in active and non-active coeliac disease. BMC Microbiol..

[58] Vrieze A., Van Nood E., Holleman F., Salojärvi J., Kootte R.S., Bartelsman J.F., Dallinga-Thie G.M., Ackermans M.T., Serlie M.J., Oozeer R. (2012). Transfer of intestinal microbiota from lean donors increases insulin sensitivity in individuals with metabolic syndrome. Gastroenterology.

[59] Ridaura V.K., Faith J.J., Rey F.E., Cheng J., Duncan A.E., Kau A.L., Griffin N.W., Lombard V., Henrissat B., Bain J.R. (2013). Gut microbiota from twins discordant for obesity modulate metabolism in mice. Science.

[60] Garrett W.S., Lord G.M., Punit S., Lugo-Villarino G., Mazmanian S.K., Ito S., Glickman J.N., Glimcher L.H. (2007). Communicable ulcerative colitis induced by T-bet deficiency in the innate immune system. Cell.

[61] Kelly C.R., Kahn S., Kashyap P., Laine L., Rubin D., Atreja A., Moore T., Wu G. (2015). Update on Fecal Microbiota Transplantation 2015: Indications, Methodologies, Mechanisms, and Outlook. Gastroenterology.

